# Dynamic analysis on simultaneous iEEG-MEG data via hidden Markov model

**DOI:** 10.1016/j.neuroimage.2021.117923

**Published:** 2021-06

**Authors:** Siqi Zhang, Chunyan Cao, Andrew Quinn, Umesh Vivekananda, Shikun Zhan, Wei Liu, Bomin Sun, Mark Woolrich, Qing Lu, Vladimir Litvak

**Affiliations:** a Key Laboratory of Child Development and Learning Science of Ministry of Education, School of Biological Sciences & Medical Engineering, Southeast University, Nanjing 210096, Jiangsu, China; b Wellcome Centre for Human Neuroimaging, UCL Institute of Neurology, 12 Queen Square, London WC1N 3BG, UK; c Department of Neurosurgery, affiliated Ruijin Hospital, Shanghai Jiao Tong University School of Medicine, Shanghai, China; d Oxford Centre for Human Brain Activity, University of Oxford, Warneford Hospital, Oxford, UK; e National Hospital for Neurology and Neurosurgery, Queen Square, London, UK

**Keywords:** Human, Dynamics, Resting state, Oscillations

## Abstract

**Background:**

Intracranial electroencephalography (iEEG) recordings are used for clinical evaluation prior to surgical resection of the focus of epileptic seizures and also provide a window into normal brain function. A major difficulty with interpreting iEEG results at the group level is inconsistent placement of electrodes between subjects making it difficult to select contacts that correspond to the same functional areas. Recent work using time delay embedded hidden Markov model (HMM) applied to magnetoencephalography (MEG) resting data revealed a distinct set of brain states with each state engaging a specific set of cortical regions. Here we use a rare group dataset with simultaneously acquired resting iEEG and MEG to test whether there is correspondence between HMM states and iEEG power changes that would allow classifying iEEG contacts into functional clusters.

**Methods:**

Simultaneous MEG-iEEG recordings were performed at rest on 11 patients with epilepsy whose intracranial electrodes were implanted for pre-surgical evaluation. Pre-processed MEG sensor data was projected to source space. Time delay embedded HMM was then applied to MEG time series. At the same time, iEEG time series were analyzed with time-frequency decomposition to obtain spectral power changes with time. To relate MEG and iEEG results, correlations were computed between HMM probability time courses of state activation and iEEG power time course from the mid contact pair for each electrode in equally spaced frequency bins and presented as correlation spectra for the respective states and iEEG channels. Association of iEEG electrodes with HMM states based on significant correlations was compared to that based on the distance to peaks in subject-specific state topographies.

**Results:**

Five HMM states were inferred from MEG. Two of them corresponded to the left and the right temporal activations and had a spectral signature primarily in the theta/alpha frequency band. All the electrodes had significant correlations with at least one of the states (*p* < 0.05 uncorrected) and for 27/50 electrodes these survived within-subject FDR correction (*q* < 0.05). These correlations peaked in the theta/alpha band. There was a highly significant dependence between the association of states and electrodes based on functional correlations and that based on spatial proximity (*p* = 5.6e^−6,^χ^2^ test for independence). Despite the potentially atypical functional anatomy and physiological abnormalities related to epilepsy, HMM model estimated from the patient group was very similar to that estimated from healthy subjects.

**Conclusion:**

Epilepsy does not preclude HMM analysis of interictal data. The resulting group functional states are highly similar to those reported for healthy controls. Power changes recorded with iEEG correlate with HMM state time courses in the alpha-theta band and the presence of this correlation can be related to the spatial location of electrode contacts close to the individual peaks of the corresponding state topographies. Thus, the hypothesized relation between iEEG contacts and HMM states exists and HMM could be further explored as a method for identifying comparable iEEG channels across subjects for the purposes of group analysis.

## Highlights

• We applied Hidden Markov Model (HMM) to concurrent MEG and intracranial EEG (iEEG).
• Epilepsy cohort HMM analysis yielded states similar to those of healthy subjects.
• iEEG power correlated with the time course of HMM states.
• Functional clusters of iEEG electrodes agreed with those based on spatial location.
• Our pipeline can be used for group analysis of concurrent MEG and invasive data.

## 1 Introduction

Prior to surgical resection of the epileptic focus, intracranial electroencephalography (iEEG) recordings are often used to guide surgical planning in patients with focal refractory epilepsy (Assi et al., 2019). The procedure involving implantation of electrodes into the brain is planned on the clinical grounds, but the invasive recording provides a window for looking into brain function with excellent spatial specificity (He et al., 2019). While iEEG is associated with sparse spatial sampling due to the limited number of electrodes implanted (Velmurugan et al., 2019), magnetoencephalography (MEG) has been an increasingly utilized non-invasive method in surgical pre-evaluation of focal epilepsy, providing high temporal and spatial resolution and a whole-brain context (Gavaret et al., 2016) to abnormal epileptic activity. When acquired in parallel, the two recording modalities offer both additional clinical insights and unique research opportunities.

Combined MEG and iEEG recordings performed at different time points showed that MEG could non-invasively identify regional interictal networks (Stefan and Trinka, 2017). iEEG implantation guided by MEG findings increases the likelihood of successful resection (Murakami et al., 2016). Several studies used acquisitions of both iEEG and MEG to explore the accuracy of MEG for localizing the epileptic focus (Kim et al., 2016), the contribution of MEG for identifying iEEG implantation sites (Agirre-Arrizubieta et al., 2014) and the correspondence between MEG and iEEG in identifying the presumed epilepsy focus (Grova et al., 2016). When directly comparing iEEG and MEG, both revealed similar propagation patterns of interictal discharges (Malinowska et al., 2014). Comparing localization results for epileptic spikes and oscillations between the two modalities showed better concordance for spikes (Jmail et al., 2016). In addition to the clinical application in epilepsy, other studies used this multimodal approach to investigate spatiotemporal profiles of word processing (McDonald et al., 2010) and the relationships of fast- and slow-timescale brain oscillatory dynamics (Zhigalov et al., 2015).

For most of these studies, the recordings were performed separately for each modality, partly due to the technical difficulty associated with acquiring simultaneous multimodal brain recordings (Dalal et al., 2009). Thus, the relationship between neural oscillations recorded at various scales could not be captured. Simultaneous recordings make it possible to explore the consistency between modalities, when the exact same brain states are assessed by both (He et al., 2019). For instance, Kakisaka et al evaluated the relationship between the spike amplitude recorded from iEEG electrodes in the lateral temporal region, and their distance from the MEG-modelled spikes (Kakisaka et al., 2012). Recently, it was shown using simultaneous recordings that both MEG and iEEG could detect epileptogenic activity from deep sources such as amygdala and hippocampus (Pizzo et al., 2019).

Although iEEG and MEG are based on different physical principles, they are both neurophysiological recording techniques which are thought to capture the same type of brain activity (Dubarry et al., 2014). Previous electrophysiological studies have revealed that resting state activity is underpinned by rich spatiotemporal dynamics (Brookes et al., 2014; O'Neill et al., 2018). In past reports on simultaneous recordings of iEEG and MEG, these temporal dynamics have not been addressed. Previously, dynamics could be characterized using time-varying measures of interactions (Chang et al., 2013; Zhang et al., 2018). But analyses using sliding time-window approaches on both resting data (de Pasquale et al., 2012) and task data (O'Neill et al., 2017) still have the problem of determining the window length. One method to define resting state networks without pre-specification of the sliding window length is Hidden Markov model (HMM). This method was shown to be able to infer a number of discrete brain states that recur at different points in time on a sub-second temporal scale (Baker et al., 2014). Each state is characterized by a certain signature, which contains spatial and (depending on the choice of HMM variant) spectral information (Vidaurre et al., 2016). Although HMM analysis was first developed for MEG, it is conceptually similar to an older approach developed for EEG called ‘microstate analysis’ (Michel and Koenig, 2018). Microstates are time epochs where EEG scalp topography remains stable for periods of around 100ms with sharp and short transitions to a different topography i.e. the next state. Segmentation of EEG scalp maps into microstates is based on finding repeating topographical patterns across multiple time points and subjects (Lehmann et al., 1987). Unlike microstate analysis, HMM explicitly models the temporal dynamics and is, therefore, tuned to finding states that repeat in a predictable way. It has been argued, however, that HMM also loses information about long-range temporal dependencies between state occurrences (Gschwind et al., 2015). In line with this, a direct comparison of HMM with microstate analysis applied to the same EEG data revealed both similarities and differences in the results (Rukat et al., 2016).Whether and how HMM states found in MEG manifest in invasive recordings is not known.

A major disadvantage of iEEG recorded in isolation is its sparse spatial sampling which is not consistent across patients. In every patient, the number of electrodes and their exact targets are determined based on the clinical presentation and pre-operative imaging. This is in contrast to electrode implantation for Deep Brain Stimulation treatment where the leads are implanted in the same target in sufficiently large patient cohorts to enable group analysis for research purposes (Holl et al., 2010). If the iEEG channels could be assigned to functional clusters based on their activity being correlated with one of the states identified with HMM, this would provide a potential way to overcome this limitation.

Here we apply a group-level HMM analysis to MEG data recorded simultaneously with iEEG in epilepsy patients at rest. Our aim was to provide a proof of principle for functional grouping of iEEG channels as described above. To this end, we aimed to show that HMM analysis for MEG is possible in this patient population despite their possibly abnormal and inconsistent functional anatomy and in the presence of interictal epileptiform activity. In addition, we wanted to test whether activity detected with iEEG can be related to HMM states identified with MEG and whether such a functional relation is consistent with the anatomical proximity between the intracranial contacts and cortical areas associated with the corresponding state.

## 2 Methods

### 2.1 Participants

Simultaneous MEG-iEEG recordings were performed on 11 patients with intractable epilepsy undergoing pre-surgery evaluations. The patients were recruited from the Department of Neurosurgery, affiliated Ruijin Hospital, Shanghai Jiao Tong University School of Medicine. Intracranial electrodes were implanted for pre-resection seizure localization guided strictly by clinical indications.

### 2.2 Ethics statement

The study was approved by the local ethics committee of Ruijin hospital, Shanghai Jiaotong University School of Medicine and in accordance with The Code of Ethics of the World Medical Association (Declaration of Helsinki) for experiments involving humans. Every patient was informed about the aim and the scope of the study and gave written informed consent.

### 2.3 Data acquisition

Implantation of the depth electrodes (SDE-08: S8 and S16, Beijing Sinovation Medical Technology CO., LTD, Beijing, China) was performed under general anaesthesia. iEEG electrodes were implanted using the orthogonal method aided by Leksell head frame. The electrodes had 8 or 16 contacts. The length of each contact was 2 mm, the distance between contacts was 1.5 mm, the contact diameter was 0.8 mm. Location and number of iEEG electrodes implanted varied between patients depending on presumed epileptogenic focus. Table 1 summarizes the patients’ clinical and iEEG characteristics. Resting MEG recordings were carried out using the Elekta Neuromag Vector View 306 channel System in a magnetically shielded chamber. The EEG system integrated with the MEG was used for the simultaneous acquisition of iEEG recordings. The sampling rate was 1000 Hz. The patients were instructed to rest with eyes closed.

**Table 1:** Clinical and iEEG characteristics of epilepsy patients.

Case	Age (years)	Gender (M/F)	Epilepsy duration (Years)	Locations of electrodes	#Contacts #Electrodes
1	24	F	2	r temp, l temp, r parietal, l parietal	32/4
2	55	F	15	l temp, r temp	16/2
3	47	F	10	r hippo, r temp, r parietal	32/4
4	14	M	6	r front, r parietal, r insular	32/4
5	33	F	14	l temp, l parietal, l hippo	32/4
6	24	F	6	r temp, r parietal	24/3
7	26	F	2	l temp, l insular, l occip, r temp	48/5
8	19	F	10	l temp, l parietal, l occip, r temp	48/6
9	33	M	9	l temp, l insula, l parietal, l front	64/6
10	32	F	14	l temp, l front	48/4
11	27	F	10	l temp, l lingual, l occip, l parietal, r hippo	64/8

### 2.4 Code and data availability statement

The code used for the analysis is available at https://github.com/SiqiZhang0106/Dynamic-HMM-Analysis-on-Simultaneous-iEEG-MEG. Data sharing is subject to ethics restrictions and therefore the data will be shared on request addressed to Dr. Chunyan Cao (chunyan_c@tongji.edu.cn) and subject to data sharing agreement.

### 2.5 Data analysis

Anatomical data were processed with the Lead-DBS toolbox (http://www.lead-dbs.org/) (Horn and Kuhn, 2015) to reconstruct the contact locations. iEEG contact locations were obtained by fusing a post-operative CT scan with a pre-operative T1 structural MRI scan and manually fitting electrode models to the artifacts seen in the CT. The electrode locations were then transformed to standard stereotactic space used in Lead DBS (MNI 2009b NLIN asymmetric template).

The MEG data were de-noised using Maxfilter^TM^ software implementing the temporal extension of the signal space separation method (tSSS) (Taulu and Hari, 2009). Interictal spikes were identified by a trained clinician and segments of ±1sec around spikes were excluded from analysis. The subsequent analyses were performed using the Oxford Centre for Human Brain Activity (OHBA) Software Library (OSL) (https://ohba-analysis.github.io/osl-docs/) (Quinn et al., 2018). This builds upon Fieldtrip (http://www.fieldtriptoolbox.org/) (Oostenveld et al., 2011) and SPM (http://www.fil.ion.ucl.ac.uk/spm/) toolboxes (Litvak et al., 2011). Structural MRI and the MEG data were co-registered by RHINO (Registration of Headshapes Including Nose) in OSL. The MEG data were then down-sampled to 250 Hz and filtered to the frequency band from 1 to 45 Hz. Time segments containing artifacts were detected using the generalized extreme studentized deviate method (Rosner, 1983) to reject outliers in the standard deviation of the signal computed across all sensors. Subsequently, temporal independent component analysis (ICA) produced independent components that were visually checked to remove artifacts related to breath, heart beats, movement and muscle activity (4.3 ± 2.1 components (mean ± SD) were removed from each data session). A Linearly Constrained Minimum Variance (LCMV) vector beamformer was applied on the pre-processed sensor data to project them onto an 8 mm grid in source space (Van Veen et al., 1997; Woolrich et al., 2011). Parcel-wise time series with 39 regions covering the entire cortex were estimated by taking the first component of a weighted Principal Component Analysis (PCA) across voxels within each parcel (Quinn et al., 2018). There were 283.2 voxels on average per parcel and the first component accounted for the majority of the variance of the voxels within the parcel (81.6% on average). A multivariate symmetric orthogonalization was then adopted to attenuate the spatial leakage effects (Colclough et al., 2015) including those caused by ghost interactions (Palva et al., 2018).

### 2.6 HMM model

**Fig. 1:**
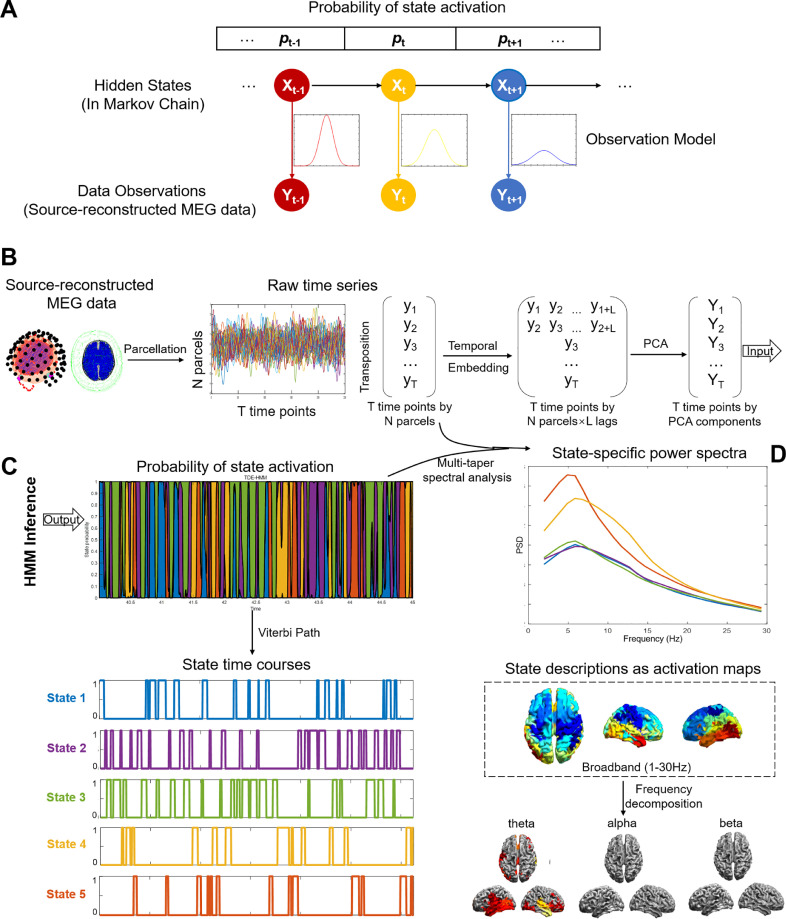
The workflow for time delay embedded HMM training and inference on MEG data. A. The schematic of a Hidden Markov Model. The basic principle assumes that a time series can be described using a hidden sequence of a finite number of states. The model then assumes that the data observed in each state are drawn from a probabilistic observation model. B. Input to HMM: Source-reconstructed MEG data were parcellated to N parcels (*N* = 39) and then concatenated across subjects. An HMM observation model was trained on an “embedding” transformation of the original data. C. Output and display of HMM results. HMM provided state probability time courses indicating the probability of each state to be active at each time point and the binary state time courses indicating the most probable sequence of states. D. The states can be further interrogated by looking at their associated power spectra and topographies for the whole analysis band (1–30 Hz) and sub-bands.

The basic principle of HMM assumes that a time series can be described using a hidden sequence of a finite number of states (Vidaurre et al., 2017) as shown in Fig. 1A. The HMM is a probabilistic model and aims to discover these hidden brain states as well as the likely sequence of transitions between them. At each time point, only one state is active, the probability of a state being active at time point t is modelled to be dependent on which state was active at time point t−1 (i.e. it is order-one Markovian) (Vidaurre et al., 2018b). The link between these hidden states and our observed data comes from an observation model (also known as emission probabilities or output probabilities) (Quinn et al., 2018). In our case, the data observation is the source-reconstructed MEG time series for a set of 39 cortical parcels (see above). The model then assumes that the data observed in each state are drawn from the probabilistic observation model. In summary, HMM infers an observation distribution corresponding to a hidden state and assigns a probability of being active to each state at each time point. For time delay embedded HMM (TDE-HMM) proposed by (Vidaurre et al., 2018b), the state observation models are characterized by multivariate auto-covariance matrices.

### 2.7 HMM inputs

The TDE-HMM is trained on the source-space MEG data using the HMM-MAR toolbox (https://github.com/OHBA-analysis/HMM-MAR). To avoid overfitting problems, sequential temporal embedding and PCA are applied to raw time series in source space, prior to training an autocovariance observation model for TDE-HMM (Vidaurre et al., 2018b). As shown in Fig. 1B, the time course of each parcel was embedded with a time delay using L lags. L was set to be 15 following (Quinn et al., 2018) with lags between −7 and 7 time steps. As we had previously down-sampled the data to 250 Hz, L of 15 corresponded to 30 ms lags in both directions and resulted for each subject in an extended data matrix of (L lags * N nodes) * S time samples. The first dimension of the matrix was reduced from 15 × N to 4 × N by principal component analysis. Time series of the components were then used for HMM training. The HMM-MAR uses stochastic inference (Vidaurre et al., 2018a) with the batch size set to use 15 continuous data segments at each iteration. This was done by taking subsets or batches of subjects at each iteration instead of the entire data set. The maximum number of variational inference cycles was set to 500. The whole HMM training procedure was repeated 10 times to ensure stability of the results and the best performance with lowest free energy was accepted.

### 2.8 HMM outputs

An observation model and a probability matrix of state activation were directly output from HMM. The state observation models were characterized by multivariate auto-covariance matrices, based on which a time series of posterior probabilities were inferred to represent the occurrence probability of a state at a time point. Importantly, an observation model trained on one data set can be applied to another dataset to directly compute state time courses and topographies. In the present study, an observation model trained on healthy subjects was applied to patients in order to compare the results to HMM estimation from the patient data.

The probability time series represent the probability of a state being active at a time point. These probabilities sum to 1 across states. The Viterbi path algorithm (Bishop, 2006) can then be applied to compute the most probable sequence of hard-assigned, i.e. non-probabilistic states. For each state, it is a binary time series representing whether the state occurs at a particular time point or not. This is conceptually different from the probability time series where for each time point more than one state can have non-zero probability. The sampling rate of both probability time series and binary time series was the same as of the raw data (250 Hz). The binary time series were only used for characterizing the states in terms of their temporal properties and the probability time series were used for computing state spectra and for correlation with iEEG power.

For each inferred state, corresponding probability time course was computed and the state-specific MEG power spectra (Fig. 1D) were estimated in the range of 1–30 Hz using state-wise multi-taper approach introduced in (Vidaurre, Quinn et al. 2016). To aid visualization, spectral modes were then generated by computing a Non-Negative Matrix Factorisation (NNMF) across the spectral estimates (Quinn et al., 2018).

### 2.9 Establishing the relation to iEEG data

**Fig. 2:**
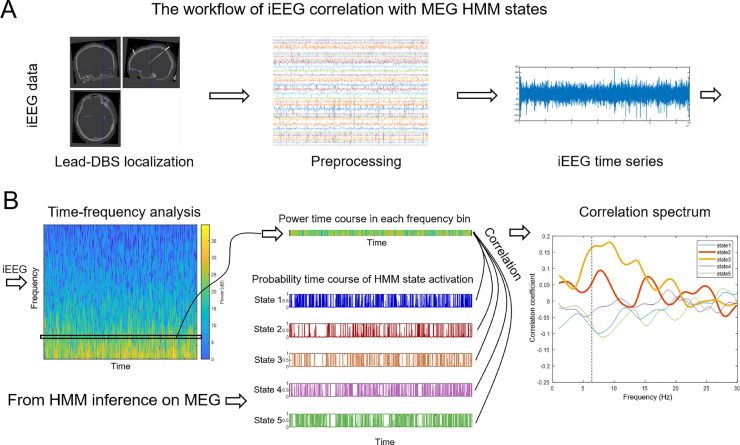
The workflow of iEEG correlation with MEG HMM states. A. iEEG contacts localization and signal preprocessing steps for the iEEG time series. B. Power time course in each frequency bin could be extracted from the iEEG time-frequency matrix. The probability matrix of HMM state activation derived from MEG had the same temporal resolution. Pearson correlation coefficients were computed between probability time courses and iEEG power time courses for each frequency bin separately and presented as correlation spectra. For each electrode there were 5 spectra (one per HMM state). The dashed line corresponds to the frequency shown by the box on the left subplot.

To interrogate the simultaneously acquired intracranial data, a ‘correlation spectrum’ was computed for each electrode's middle bipolar channel (4–5 for 8-contact electrodes, 8–9 for 16 contacts) and each MEG-derived HMM state. We will henceforth refer to the middle channel as ‘electrode’ for clarity. This procedure was done as shown in Fig. 2. First, time-frequency decomposition was calculated for each electrode with the same time resolution as MEG HMM probability time course. This afforded sample-by-sample correspondence between HMM probability time courses and iEEG power time courses. Pearson correlation coefficients were then computed between each HMM-derived state probability time course and the iEEG power time series for each frequency and plotted as a function of frequency, resulting in a separate correlation spectrum for each combination of HMM state and electrode.

To test whether there was a correspondence between functional correlation of iEEG power with HMM states and the spatial location of the corresponding electrodes, we compared the assignment of electrodes to states based on those two criteria. The criterion for functional assignment was that the correlation was significant (*p* < 0.05, uncorrected) and higher than that for the other states. The criterion for spatial assignment was the smallest mean Euclidian distance between the midpoint of the two iEEG contacts and the voxels in the most activated cluster for a particular state. This cluster was defined by thresholding the patient-specific state topography at 90% and taking the blob with the largest number of voxels. χ^2^ test for independence of categorical variables (*crosstab* function in MATLAB) was used to test for significance of agreement between the two ways of assignment.

### 2.10 Comparison to HMM derived from a healthy subject dataset

Although epochs of abnormal interictal activity were removed, slow wave activity associated with epilepsy could possibly still affect the MEG-derived HMM state models. Thus, the HMM states derived from the patient MEG data were validated by using an HMM model trained on a large number of healthy subjects acquired as part of a different project at OHBA. This healthy dataset was also recorded in a Neuromag 306 MEG system, and its pre-processing and TDE-HMM training were done in the same way as for our patient analysis. We then sought to estimate the state-specific spectral group activation maps in the patient MEG that corresponded to the HMM states inferred on the healthy cohort. First, we extracted the state-specific group observation models (that is, the HMM structure in Fig 1A) from HMM trained on the healthy dataset. Then we applied this structure to the patient data and repeated the procedures of Fig. 1C and D to compute the state probability time courses and the state spectra and topographies. Based on the state probability courses inferred from the healthy HMM, the state-specific spectral activation group maps were estimated for the patient MEG data.

## 3 Results

Data from 11 patients were included in the analysis. The duration of resting-state recordings was 397.90 ± 125.56 s. The differences in duration between patients were due to clinical constraints. The length of data removed to exclude interictal spikes was 11.27 ± 9.96 s across all patients. The breakdown by patient is shown in Supplementary Fig. S1.

### 3.1 HMM results from MEG data

**Fig. 3:**
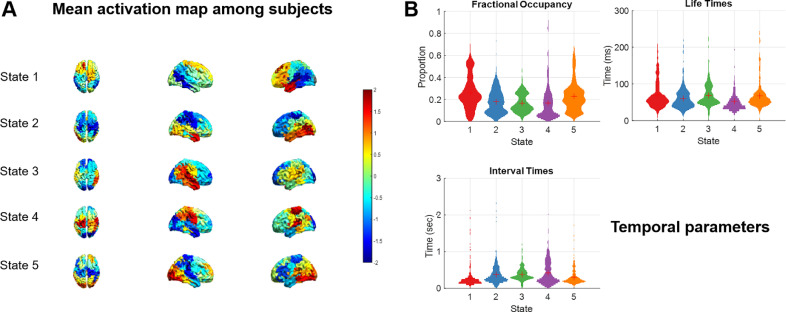
Outputs from running HMM on the MEG data. A. Mean spectral activation maps of five states computed across all subjects in the broad (1–30 Hz) band. See supplementary Fig. S3 for comparison with individual topographies. B. Distribution of temporal parameters of the five states: fractional occupancy, life times and interval times.

After extracting and concatenating MEG data of 11 subjects, we identified 5 HMM states using TDE-HMM. Prior to that we tested a range of values for K - the number of HMM states. K settings above 5 did not change the topographies of the most common states. K settings below 5 resulted in states that conflated some of the states visible for higher values of K and thus lacked clear and focal topography. The corresponding results are shown in supplementary Fig. S2. Once the HMM model was trained on the MEG data, we could obtain the state time courses and the spectral signature for each state. Fig. 3 shows spatial power maps and temporal features of all the states at the group level. Looking closely at the five mean activation maps averaged across 11 subjects, state 1 showed a large-scale activation in the fronto-parietal area in both hemispheres. States 2 and 3 corresponded to the left and the right temporal activations respectively. State 4 corresponded to the sensorimotor areas and state 5 was expressed stronger in the occipital areas. The transition matrix of HMM states can be found in supplementary Figure S4.

Three parameters: fractional occupancy, life times and interval times were used to illustrate the temporal statistics of each state. Fractional occupancy for a state is the proportion of time each subject spent in this state. The state life time refers to the number of time points per visit, known as the duration of visits to a state. This reflects the temporal stability of the states. The state interval time is the number of time points between subsequent visits. All the five states had similar fractional occupancies around 15–25%. The state life times of all the states were around 50-100ms. The state interval times were also distributed similarly.

### 3.2 Correlation of MEG-derived HMM and iEEG data

**Fig. 4:**
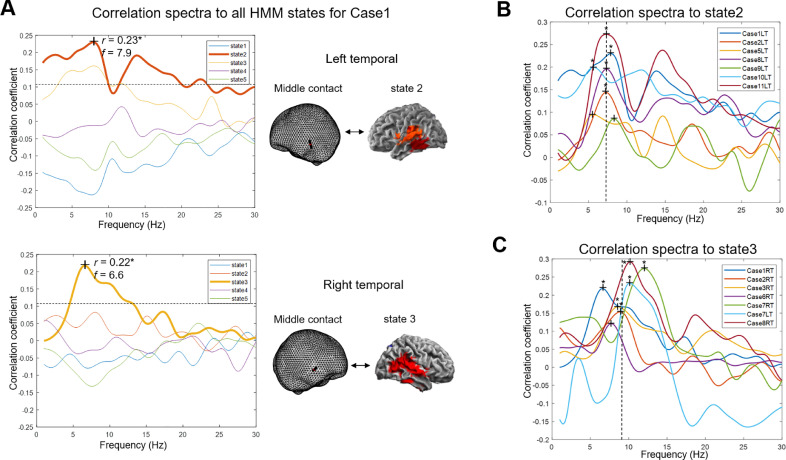
Correlation spectra of MEG-derived HMM results and iEEG data **A.** Correlation spectra for two bilateral temporal electrodes in a representative patient (Case 1). The dotted line is the minimum *r* whose *p* value survives within-patient FDR correction. **B.** Correlation spectra between the right temporal electrodes (midpoints) and **state 3** for all the right temporal implanted patients. (Case 7 left temporal included as its maximum correlation was with state 3). **C.** Correlation spectra between the left temporal electrodes (midpoints) and **state 2** for all the left temporal implanted patients. (Case 7 excluded as its maximum correlation with state 3, see B). The correlation peaks are marked with **^+^** and those surviving FDR correction are also marked with *. The dashed lines in B and C correspond to the averaged peak frequency.

The state-wise correlation spectra were computed for each iEEG electrode based on the TDE-HMM results derived from MEG data. Correlation spectrum was defined as the series of frequency-specific Pearson correlation coefficients between the probability time course of a particular MEG-derived HMM state and the power time course of the iEEG electrode in one frequency bin (see Methods and Fig. 2 for details). All the 50 electrodes had correlations with at least one of the states that were significant at the uncorrected level (*p* < 0.05) and for 27 electrodes these correlations survived within-subject FDR correction (*q* < 0.05). Most of these correlations were with the temporal region states (states 2 and 3 in Fig. 3) and we, therefore, focused on temporal regions in more detail. Ten out of the eleven patients included in the study were implanted with temporal electrodes. A typical patient with both left and right temporal iEEG electrodes is presented in Fig. 4A. The correlation between HMM probability time series of state 2 and 3 (from MEG data) and temporal spectral power (from iEEG data) was highest compared with other states in theta/alpha band with peak values *r* = 0.23, *f* = 7.9 Hz, *p* < 0.0001 (left temporal) and *r* = 0.22, *f* = 6.6 Hz, *p* < 0.0001 (right temporal). Supplementary Table 1 reports peak correlations for all patients, electrodes and states.

**Fig. 5:**
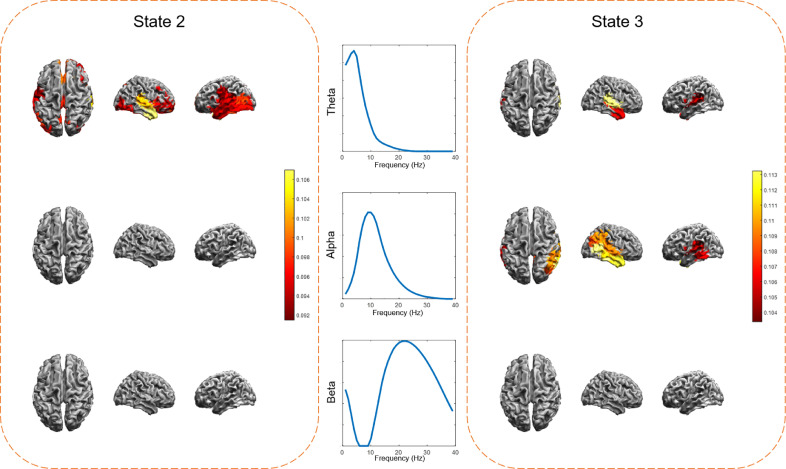
Spectral decomposition of two MEG HMM states. The band-limited power maps for state 2 and state 3 showed that the majority of their power concentrated in the theta and alpha frequencies in the temporal cortex which is consistent with the correlation spectra peak frequencies (see Fig. 4).

For all ten patients with temporal electrodes, power recorded from temporal channels had consistently high correlations with the right and the left temporal HMM states as shown in Fig. 4B and 4C. Maximum correlation values with the left temporal HMM state (state 2) were usually found in the theta band, and maximum correlation values with the right temporal HMM state (state 3) were usually found in the alpha band. Interestingly, spectrally resolved power maps of the two temporal states (Fig. 5) showed that States 2 and 3 were characterized by increased theta and alpha power in the left and right temporal areas respectively, consistent with the frequency range of the highest correlations. The individual results, however, did not always follow the group patterns (e.g. Fig. 4A (state 3), see also Discussion).

**Fig. 6:**
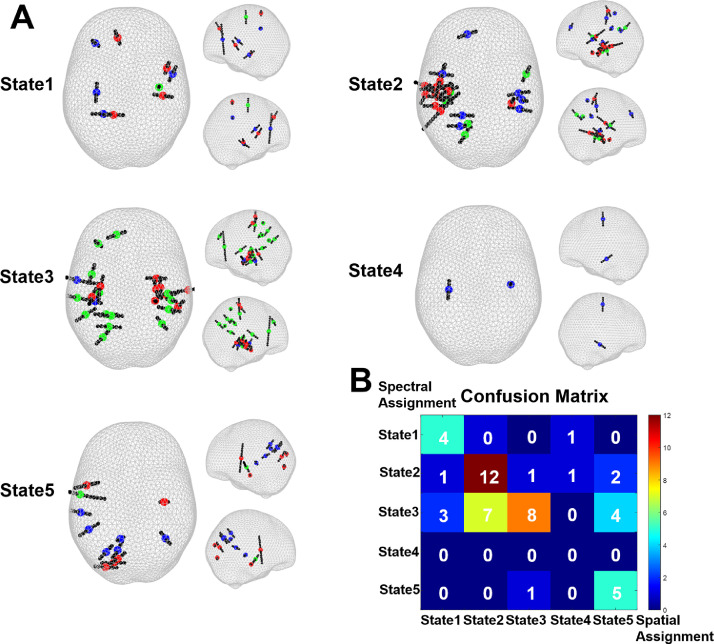
Comparison between the two methods of assigning electrodes to HMM states (spatial proximity vs. functional correlations). **A.** Allocation of electrodes to states is shown for each state separately. The mid contacts of electrodes assigned to the same state by both methods are in red, only spatial are in blue and only spectral are in green. Note that the spatial assignment is based on individual topographies and therefore might not be consistent with the group maps shown in Fig. 3. **B.** The confusion matrix of the two assignment methods. There was agreement for 29/50 electrodes i.e. 58%, (*p* = 5.6e^−6^, χ^2^ test for independence).

To test whether there was correspondence between functional correlation of iEEG power with HMM states and the spatial location of the corresponding electrodes we compared the assignment of electrodes to states based on those two criteria (Fig. 6, see Methods for details). The most frequently assigned states for all contacts were state 2 and state 3, which corresponded to the locations of contacts in the temporal lobes being more common for all patients than elsewhere. There was good concordance between the two criteria with agreement for 29/50 electrodes i.e. 58%, (*p* = 5.6e^−6^, χ^2^ test for independence). Note, however, that neither of the two methods can be considered the ground truth (see further in the Discussion).

**Fig. 7:**
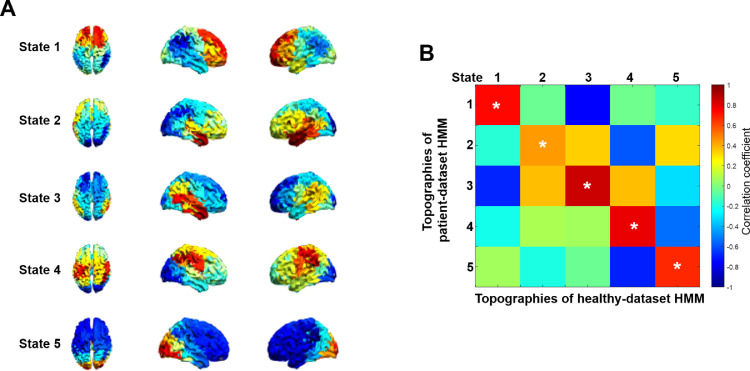
Validation of the patient MEG HMM inference using HMM trained on a MEG dataset of healthy subjects. Note that the order of states in HMM output is arbitrary and they were reordered to match the order for the patient group. **A.** Group topographies for the epilepsy dataset estimated from the HMM model trained on a healthy-cohort. **B.** Pearson correlation coefficients between the topographies of healthy fitted HMM model and our patient data-driven HMM model (Fig. 3A). Note that the matrix is not symmetric because it shows correlations between two sets of topographies rather than within the same set.

Finally, to make sure that the HMM inference was not driven by abnormal activity related to epilepsy, we repeated the analysis using HMM observation models trained on a set of healthy subjects (see Methods). The activation maps displayed in Fig. 7A looked very similar to those acquired from HMM trained on patient MEG data as shown in Fig. 3A. The similarity was quantified by Pearson correlation between the spatial activations of healthy fitted HMM output and our data-driven HMM output and was significant with *p* < 0.01 for the corresponding states (Fig. 7B). This suggests that abnormal functional anatomy in patients does not preclude group HMM analysis.

## 4 Discussion

Non-invasive whole-brain MEG recordings could help put iEEG data in the context of overall brain activity so that both modalities would maintain their inherent advantages whilst overcoming their limitations. By means of simultaneous MEG recording, large-scale networks in resting-state brain can be well described by repeated visits to short-lived transient brain states identified with TDE-HMM. This method provides information that is both spectrally and temporally resolved as different networks are described as being active or inactive at different points in time (Vidaurre et al., 2018b). This is the first time HMM was applied to a simultaneously recorded iEEG-MEG dataset and our pipeline (https://github.com/SiqiZhang0106/Dynamic-HMM-Analysis-on-Simultaneous-iEEG-MEG) could be used in future similar studies.

Five resting-state HMM states were inferred from the MEG dataset after removal of clearly abnormal interictal activity and exhibited evenly distributed temporal characteristics. The power time courses of iEEG electrodes were correlated with HMM state probability time courses and there was concordance between assignment of electrodes to states based on these correlations and the assignment based on spatial proximity. We consider it as evidence for our hypothesis that HMM applied to MEG can be used for functional grouping of simultaneously recorded iEEG channels. There are, however, several limitations to the study and we will discuss them below.

The sample size was limited because of the technical difficulty of acquiring simultaneous iEEG and MEG data although our dataset was quite large compared to other similar studies (Badier et al., 2017; Dalal et al., 2013). Most patients (10 out of 11) were implanted with temporal electrodes and showed correlations primarily with the temporal states 2 and 3. No electrodes at all were assigned to state 4 based on the functional correlations and only a few were assigned to state 5. It would be preferable to run this analysis on a dataset with more complete and uniform coverage of the brain but there are several factors making this difficult. Medial temporal lobe, particularly the areas around the amygdala and the hippocampus, are common locations of seizure onset and, therefore, more frequently targeted (Irena et al., 2017). The small number of centers that do this kind of recordings makes it difficult to create a more comprehensive multi-center dataset as has been done for intracranial recordings alone (Trebaul et al., 2018).

We only looked at the middle iEEG channel for every electrode, leaving large part of the data unexplored. That simplified the presentation for this proof-of-principle report and reduced the number of multiple comparisons. For a more comprehensive analysis, looking at all the channels will be required as they might span several different cortical regions. It might be necessary to devise a statistical method that will take into account the spatial arrangement of the contacts and be sensitive to significant clusters spanning several adjacent channels to improve the statistical sensitivity with increased number of multiple correlations.

Although some of the subject-specific state topographies were highly similar to the group topographies, this was not always the case. In one case as shown in Fig. 4, the individual peak was even contralateral to the group peak. This is not surprising in light of the individual variability in functional anatomy which might be even greater in patients due to cortical dysfunction (Lado et al., 2002; Springer et al., 1999). The whole idea of using MEG-derived HMM as suggested in the present study is that it would account for this and our results indeed show is that this variability exists and can be seen with both the non-invasive and the invasive approach.

While we took care to exclude data segments around interictal spikes from the analysis we cannot completely rule out a contribution of abnormal activity to both the pattern of MEG-derived states and to correlations with iEEG. EEG features in the theta/alpha band have been shown to differ between people with epilepsy and healthy controls (Yaakub et al., 2020). Our spectral analysis showed that the two temporal states were mostly activated in these bands and also the correlation spectra peaks were primarily found there. However, using an observation model derived from a healthy subject cohort did not substantially alter the observed pattern of states. Furthermore, each cortical area tended to preserve its own natural frequency, indicating that the observed oscillations reflect local physiological mechanisms (Rosanova et al., 2009). Thus, we have reasons to believe that the activity driving our results is primarily non-pathological but further research is necessary to address this in a conclusive way.

The brain template we used in the parcellation of MEG data is a cortical template as in the previous HMM studies (Quinn et al., 2018). We, therefore, did not address the subcortical dynamics with our HMM analysis. Even with a different parcellation it would be difficult to do it with MEG due to its low sensitivity to deep-source signals. Although it was recently shown that hippocampus and amygdala contribute to MEG signals (Pizzo et al., 2019), it is not clear whether this contribution is sufficient to drive HMM state changes. So it could well be the case that the kind of correlation analysis shown here would not work for some of the deep structures. This issue could be addressed by doing a more detailed anatomical analysis of contact locations that is beyond the scope of the present study but would be the next logical step.

Another factor possibly contributing to low HMM-iEEG correlations is the fact that some of the states (like the fronto-parietal state 1 in our analysis) lump together very large and different cortical areas whereas the sensitivity profile of iEEG is much more focal. This might explain why although the parietal electrodes in our dataset were correlated stronger with state 1 than with the other states, these correlations did not survive FDR correction. For our envisioned purpose of categorizing iEEG contacts, a much more fine-grained set of states would be necessary to make it practically useful. However, with the current HMM technology and the size of our dataset, only a small number of HMM states can be identified in a robust and reproducible way. This limitation could possibly be overcome with a much larger dataset (e.g. by pooling data from different clinical sites). It could be expected that with a more fine-grained sets of states it would be possible to better account for between-subject variability. With a larger dataset, there is also the option to increase other complexities in the model (e.g. increasing the complexity of the observation model and moving beyond the Markovian assumption), and thus, more sophisticated approaches could be possible.

To validate the idea of using functional correlations to categorize iEEG electrodes, we compared this criterion to another one based on spatial proximity. However, this spatial criterion is by no means the gold standard or the ground truth. The only conclusion that can be drawn from this comparison is that there is some consistency between the location of iEEG contacts with respect to individual state topography and the correlation of iEEG activity with that state. A better validation could be made based on an individual functional parcellation e.g using resting fMRI and tractography (Glasser et al., 2016), but this kind of data was not available for our cohort.

We used the fully-connected HMM state model in the current resting state study. For task-evoked dynamic analysis in the future, a restrictive version of the HMM model may be advantageous to explore state-wise task dynamics. The HMM inference could be restricted in time to infer states only from epochs of interest within the task. An HMM whose states allow the maximum decoding of task conditions or behavioral performance could be selected in such studies. This is potentially meaningful to a range of cognitive and clinical applications. Finally, the HMM inferred in this manuscript is unsupervised with respect to resting-state condition, however the inference may be tuned to perform supervised learning in future task studies.

## 5 Conclusions

We showed for the first time that global brain states, identified using HMM from non-invasive MEG data lending itself to group analysis, have clear intracranial correlates. Although this fact is not surprising given that both MEG and iEEG data are generated by cortical activity, it forms the basis of further exploration of the possibly complex relations between the two kinds of signals using HMM methods. We also demonstrated the feasibility of functional categorization of iEEG contacts based on their correlations with HMM states. However, further technical development is necessary before this idea can be applied in practice.

## Code and data availability statement

The code used for the following analysis presented here is available at https://github.com/SiqiZhang0106/Dynamic-HMM-Analysis-on-Simultaneous-iEEG-MEG. Data sharing is subject to ethics restrictions and therefore the data will be shared on request addressed to Dr. Chunyan Cao (chunyan_c@tongji.edu.cn) and subject to data sharing agreement.

## CRediT authorship contribution statement

**Siqi Zhang:** Methodology, Software, Formal analysis, Visualization, Writing - original draft. **Chunyan Cao:** Investigation, Resources, Data curation, Funding acquisition, Writing - review & editing. **Andrew Quinn:** Methodology, Software, Validation. **Umesh Vivekananda:** Formal analysis, Investigation. **Shikun Zhan:** Resources, Data curation. **Wei Liu:** Resources, Data curation. **Bomin Sun:** Resources, Data curation. **Mark Woolrich:** Methodology, Software, Writing - review & editing. **Qing Lu:** Writing - review & editing, Supervision, Funding acquisition. **Vladimir Litvak:** Conceptualization, Methodology, Writing - review & editing.

## Declaration of Competing Interest

None.

## Appendix Supplementary materials

Image, application 1

## References

[bib0001] Agirre-Arrizubieta Z., Thai N.J., Valentín A., Furlong P.L., Seri S., Selway R.P., Elwes R.D., Alarcón G. (2014). The value of Magnetoencephalography to guide electrode implantation in epilepsy. Brain Topogr..

[bib0002] Assi E.B., Rihana S., Nguyen D.K., Sawan M. (2019). Effective connectivity analysis of iEEG and accurate localization of the epileptogenic focus at the onset of operculo-insular seizures. Epilepsy Res..

[bib0003] Badier J., Dubarry A., Gavaret M., Chen S., Trebuchon A., Marquis P., Regis J., Bartolomei F., Benar C., Carron R. (2017). Technical solutions for simultaneous MEG and SEEG recordings: towards routine clinical use. Physiol. Mea..

[bib0004] Baker A.P., Brookes M.J., Rezek I.A., Smith S.M., Behrens T., Probert Smith P.J., Woolrich M. (2014). Fast transient networks in spontaneous human brain activity. Elife.

[bib0005] Bishop C.M. (2006). Pattern Recognition and Machine Learning.

[bib0006] Brookes M.J., O'Neill G.C., Hall E.L., Woolrich M.W., Baker A., Palazzo Corner S., Robson S.E., Morris P.G., Barnes G.R. (2014). Measuring temporal, spectral and spatial changes in electrophysiological brain network connectivity. Neuroimage.

[bib0007] Chang C., Liu Z., Chen M.C., Liu X., Duyn J.H. (2013). EEG correlates of time-varying BOLD functional connectivity. Neuroimage.

[bib0008] Colclough G.L., Brookes M.J., Smith S.M., Woolrich M.W. (2015). A symmetric multivariate leakage correction for MEG connectomes. Neuroimage.

[bib0009] Dalal S.S., Baillet S., Adam C., Ducorps A., Schwartz D., Jerbi K., Bertrand O., Garnero L., Martinerie J., Lachaux J.-P. (2009). Simultaneous MEG and intracranial EEG recordings during attentive reading. Neuroimage.

[bib0010] Dalal S.S., Jerbi K., Bertrand O.F., Adam C., Ducorps A., Schwartz D., Martinerie J., Lachaux J. (2013). Simultaneous MEG-intracranial EEG: new insights into the ability of MEG to capture oscillatory modulations in the neocortex and the hippocampus. Epilepsy Behav..

[bib0011] de Pasquale F., Della Penna S., Snyder A.Z., Marzetti L., Pizzella V., Romani G.L., Corbetta M. (2012). A cortical core for dynamic integration of functional networks in the resting human brain. Neuron.

[bib0012] Dubarry A.-S., Badier J.-M., Trébuchon-Da Fonseca A., Gavaret M., Carron R., Bartolomei F., Liégeois-Chauvel C., Régis J., Chauvel P., Alario F.-X. (2014). Simultaneous recording of MEG, EEG and intracerebral EEG during visual stimulation: from feasibility to single-trial analysis. Neuroimage.

[bib0013] Gavaret M., Dubarry A.-S., Carron R., Bartolomei F., Trébuchon A., Bénar C.-G. (2016). Simultaneous SEEG-MEG-EEG recordings overcome the SEEG limited spatial sampling. Epilepsy Res..

[bib0014] Glasser M.F., Coalson T.S., Robinson E.C., Hacker C.D., Harwell J., Yacoub E., Ugurbil K., Andersson J., Beckmann C.F., Jenkinson M., Smith S.M., Van Essen D.C. (2016). A multi-modal parcellation of human cerebral cortex. Nature.

[bib0015] Grova C., Aiguabella M., Zelmann R., Lina J.M., Hall J.A., Kobayashi E. (2016). Intracranial EEG potentials estimated from MEG sources: a new approach to correlate MEG and iEEG data in epilepsy. Hum. Brain Mapp..

[bib0016] Gschwind M., Michel C.M., Van De Ville D. (2015). Long-range Dependencies Make the Difference-Comment on 'A Stochastic Model for EEG Microstate Sequence Analysis.

[bib0017] He B., Astolfi L., Valdes-Sosa P.A., Marinazzo D., Palva S.O., Benar C., Michel C.M., Koenig T. (2019). Electrophysiological brain connectivity: theory and implementation. IEEE Trans. Biomed. Eng..

[bib0018] Holl E.M., Petersen E.A., Foltynie T., Martinez-Torres I., Limousin P., Hariz M.I., Zrinzo L. (2010). Improving Targeting in Image-Guided Frame-Based Deep Brain Stimulation.

[bib0019] Horn A., Kuhn A.A. (2015). Lead-DBS: a toolbox for deep brain stimulation electrode localizations and visualizations. Neuroimage.

[bib0020] Irena D., Milan B., Philippe K. (2017). Temporal lobe epilepsy? Things are not always what they seem. Epileptic Disord..

[bib0021] Jmail N., Gavaret M., Bartolomei F., Chauvel P., Badier J., Benar C.G. (2016). Comparison of brain networks during interictal oscillations and spikes on magnetoencephalography and intracerebral EEG. Brain Topogr..

[bib0022] Kakisaka Y., Kubota Y., Wang Z.I., Piao Z., Mosher J.C., Gonzalez-Martinez J., Jin K., Alexopoulos A.V., Burgess R.C. (2012). Use of simultaneous depth and MEG recording may provide complementary information regarding the epileptogenic region. Epileptic Disord..

[bib0023] Kim D., Joo E.Y., Seo D.-W., Kim M.-Y., Lee Y.-H., Kwon H.C., Kim J.-M., Hong S.B. (2016). Accuracy of MEG in localizing irritative zone and seizure onset zone: quantitative comparison between MEG and intracranial EEG. Epilepsy Res..

[bib0024] Lado F.A., Legatt A.D., LaSala P.A., Shinnar S. (2002). Alteration of the cortical motor map in a patient with intractable focal seizures. (Short Report). J. Neurol. Neurosurg. Psychiatry.

[bib0025] Lehmann D., Ozaki H., Pal I. (1987). EEG alpha map series: brain micro-states by space-oriented adaptive segmentation. Electroencephalogr. Clin. Neurophysiol..

[bib0026] Litvak V., Mattout J., Kiebel S.J., Phillips C., Henson R.N., Kilner J.M., Barnes G.R., Oostenveld R., Daunizeau J., Flandin G. (2011). EEG and MEG data analysis in SPM8. Comput. Intell. Neurosci..

[bib0027] Malinowska U., Badier J.-M., Gavaret M., Bartolomei F., Chauvel P., Benar C.-G. (2014). Interictal Networks in Magnetoencephalography.

[bib0028] McDonald C.R., Thesen T., Carlson C., Blumberg M., Girard H.M., Trongnetrpunya A., Sherfey J.S., Devinsky O., Kuzniecky R., Dolye W.K. (2010). Multimodal imaging of repetition priming: using fMRI, MEG, and intracranial EEG to reveal spatiotemporal profiles of word processing. Neuroimage.

[bib0029] Michel C.M., Koenig T. (2018). EEG microstates as a tool for studying the temporal dynamics of whole-brain neuronal networks: a review. Neuroimage.

[bib0030] Murakami H., Wang Z.I., Marashly A., Krishnan B., Prayson R.A., Kakisaka Y., Mosher J.C., Bulacio J., Gonzalez-Martinez J.A., Bingaman W.E. (2016). Correlating Magnetoencephalography to Stereo-Electroencephalography in Patients Undergoing Epilepsy Surgery.

[bib0031] O’Neill G.C., Tewarie P., Vidaurre D., Liuzzi L., Woolrich M.W., Brookes M.J. (2018). Dynamics of large-scale electrophysiological networks: a technical review. Neuroimage.

[bib0032] O'Neill G.C., Tewarie P.K., Colclough G.L., Gascoyne L.E., Hunt B.A.E., Morris P.G., Woolrich M.W., Brookes M.J. (2017). Measurement of dynamic task related functional networks using MEG. Neuroimage.

[bib0033] Oostenveld R., Fries P., Maris E., Schoffelen J. (2011). FieldTrip: open source software for advanced analysis of MEG, EEG, and invasive electrophysiological data. Comput. Intell. Neurosci..

[bib0034] Palva J.M., Wang S.H., Palva S., Zhigalov A., Monto S., Brookes M.J., Schoffelen J.-M., Jerbi K. (2018). Ghost interactions in MEG/EEG source space: a note of caution on inter-areal coupling measures. Neuroimage.

[bib0035] Pizzo F., Roehri N., Villalon S.M., Trebuchon A., Chen S., Lagarde S., Carron R., Gavaret M., Giusiano B., Mcgonigal A. (2019). Deep brain activities can be detected with magnetoencephalography. Nat. Commun..

[bib0036] Quinn A.J., Vidaurre D., Abeysuriya R., Becker R., Nobre A.C., Woolrich M.W. (2018). Task-evoked dynamic network analysis through hidden Markov modeling. Front. Neurosci..

[bib0037] Rosanova M., Casali A.G., Bellina V., Resta F., Mariotti M., Massimini M. (2009). Natural frequencies of human corticothalamic circuits. J. Neurosci..

[bib0038] Rosner B. (1983). Percentage points for a generalized ESD many-outlier procedure. Technometrics.

[bib0039] Rukat T., Baker A., Quinn A., Woolrich M. (2016). Resting State Brain Networks From EEG: Hidden Markov States vs. Classical Microstates.

[bib0040] Springer J.A., Binder J.R., Hammeke T.A., Swanson S.J., Frost J.A., Bellgowan P.S.F., Brewer C.C., Perry H.M., Morris G.L., Mueller W.M. (1999). Language Dominance in Neurologically Normal and Epilepsy Subjects. A Functional MRI Study.

[bib0041] Stefan H., Trinka E. (2017). Magnetoencephalography (MEG): Past, current and future perspectives for improved differentiation and treatment of epilepsies. Seizure.

[bib0042] Taulu S., Hari R. (2009). Removal of magnetoencephalographic artifacts with temporal signal-space separation: demonstration with single-trial auditory-evoked responses. Hum. Brain Mapp..

[bib0043] Trebaul L., Deman P., Tuyisenge V., Jedynak M., Hugues E., Rudrauf D., Bhattacharjee M., Tadel F., Chanteloup-Foret B., Saubat C., Reyes Mejia G.C., Adam C., Nica A., Pail M., Dubeau F., Rheims S., Trébuchon A., Wang H., Liu S., Blauwblomme T., Garcés M., De Palma L., Valentin A., Metsähonkala E.-L., Petrescu A.M., Landré E., Szurhaj W., Hirsch E., Valton L., Rocamora R., Schulze-Bonhage A., Mindruta I., Francione S., Maillard L., Taussig D., Kahane P., David O. (2018). Probabilistic functional tractography of the human cortex revisited. Neuroimage.

[bib0044] Van Veen B.D., Van Drongelen W., Yuchtman M., Suzuki A. (1997). Localization of brain electrical activity via linearly constrained minimum variance spatial filtering. IEEE Trans. Biomed. Eng..

[bib0045] Velmurugan J., Nagarajan S.S., Mariyappa N., Mundlamuri R.C., Raghavendra K., Bharath R.D., Saini J., Arivazhagan A., Rajeswaran J., Mahadevan A. (2019). Magnetoencephalography imaging of high frequency oscillations strengthens presurgical localization and outcome prediction. Brain.

[bib0046] Vidaurre D., Abeysuriya R., Becker R., Quinn A.J., Alfaro-Almagro F., Smith S.M., Woolrich M.W. (2018). Discovering dynamic brain networks from big data in rest and task. Neuroimage.

[bib0047] Vidaurre D., Hunt L.T., Quinn A.J., Hunt B.A., Brookes M.J., Nobre A.C., Woolrich M.W. (2018). Spontaneous cortical activity transiently organises into frequency specific phase-coupling networks. Nat. Commun..

[bib0048] Vidaurre D., Quinn A.J., Baker A.P., Dupret D., Tejero-Cantero A., Woolrich M.W. (2016). Spectrally resolved fast transient brain states in electrophysiological data. Neuroimage.

[bib0049] Vidaurre D., Smith Stephen M., Woolrich Mark W. (2017). Brain network dynamics are hierarchically organized in time. Proc. Natl. Acad. Sci. US A.

[bib0050] Woolrich M., Hunt L., Groves A., Barnes G. (2011). MEG beamforming using Bayesian PCA for adaptive data covariance matrix regularization. Neuroimage.

[bib0051] Yaakub S.N., Tangwiriyasakul C., Abela E., Koutroumanidis M., Elwes R.D.C., Barker G.J., Richardson M.P. (2020). Heritability of alpha and sensorimotor network changes in temporal lobe epilepsy. Ann. Clin. Transl. Neurol..

[bib0052] Zhang S., Tian S., Chattun M.R., Tang H., Yan R., Bi K., Yao Z., Lu Q. (2018). A supplementary functional connectivity microstate attached to the default mode network in depression revealed by resting-state magnetoencephalography. Prog. Neuro-psychopharmacol. Biol. Psychiatry.

[bib0053] Zhigalov A., Arnulfo G., Nobili L., Palva S., Palva J.M. (2015). Relationship of fast-and slow-timescale neuronal dynamics in human MEG and SEEG. J. Neurosci..

