# Supplementary material for: Dynamic analysis on simultaneous iEEG-MEG data via hidden Markov model
Source: Neuroimage. 2021 Jun;233:117923. doi: 10.1016/j.neuroimage.2021.117923 (PMC8204269; doi:10.1016/j.neuroimage.2021.117923)
Supplement: Supplementary file 1 [file mmc1.docx]

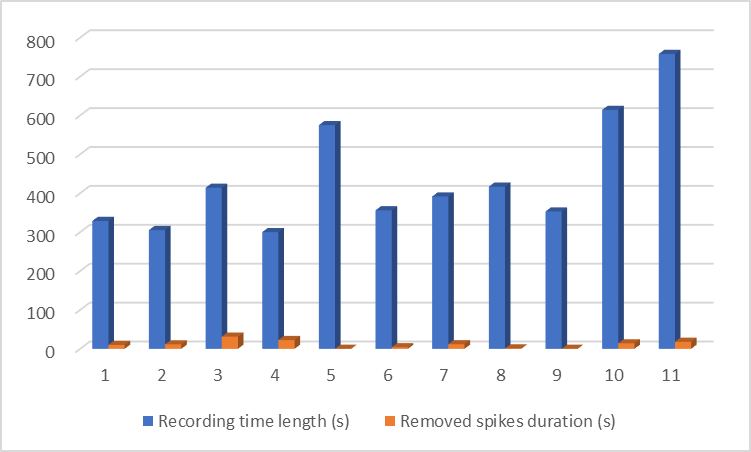


Supplementary Figure S1. The duration of resting-state recordings and removed spikes duration for each patient.


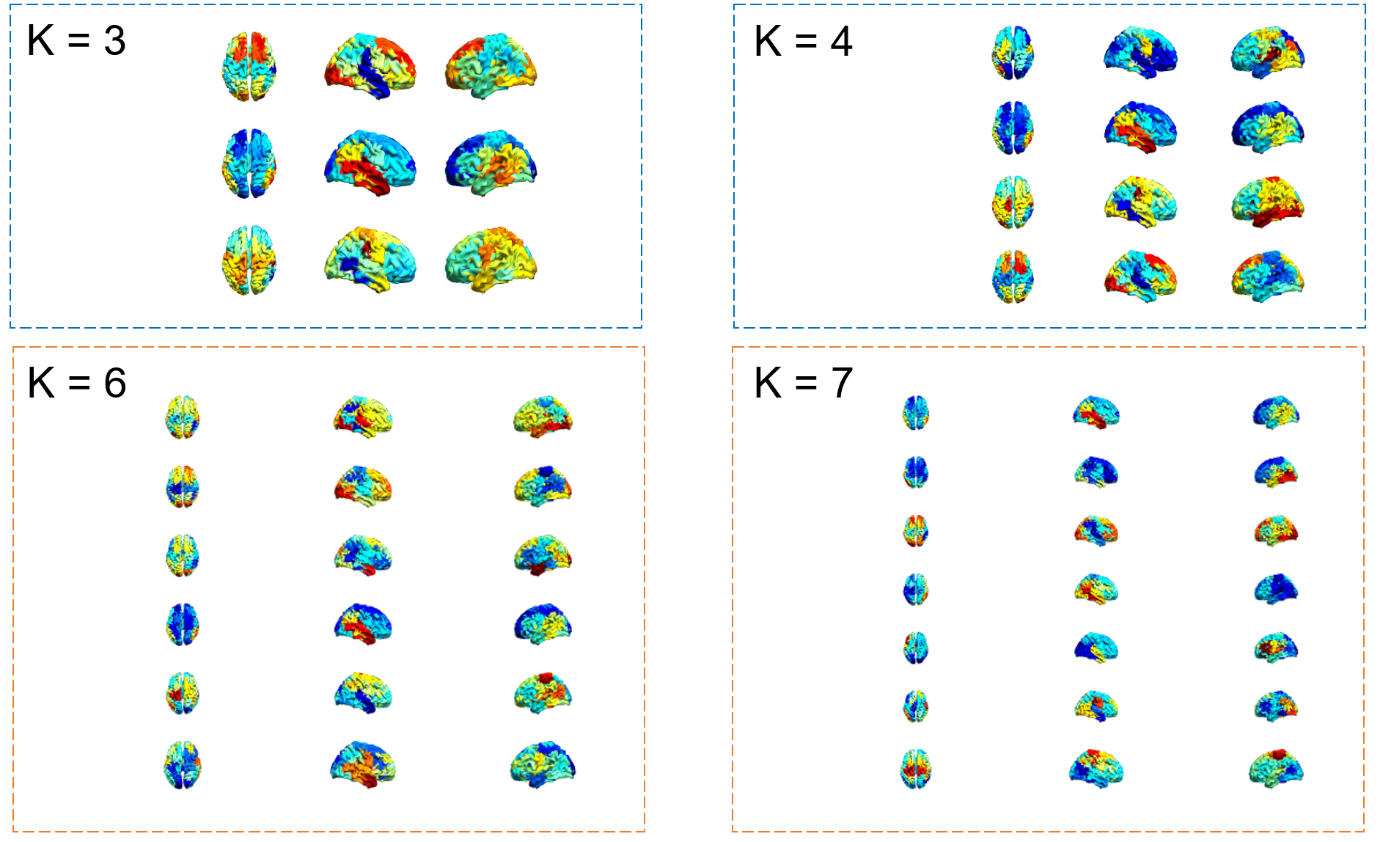


Supplementary Figure S2. Mean activation maps for HMM state numbers K from 3 to 7 (K=5 can be found in Figure 3)


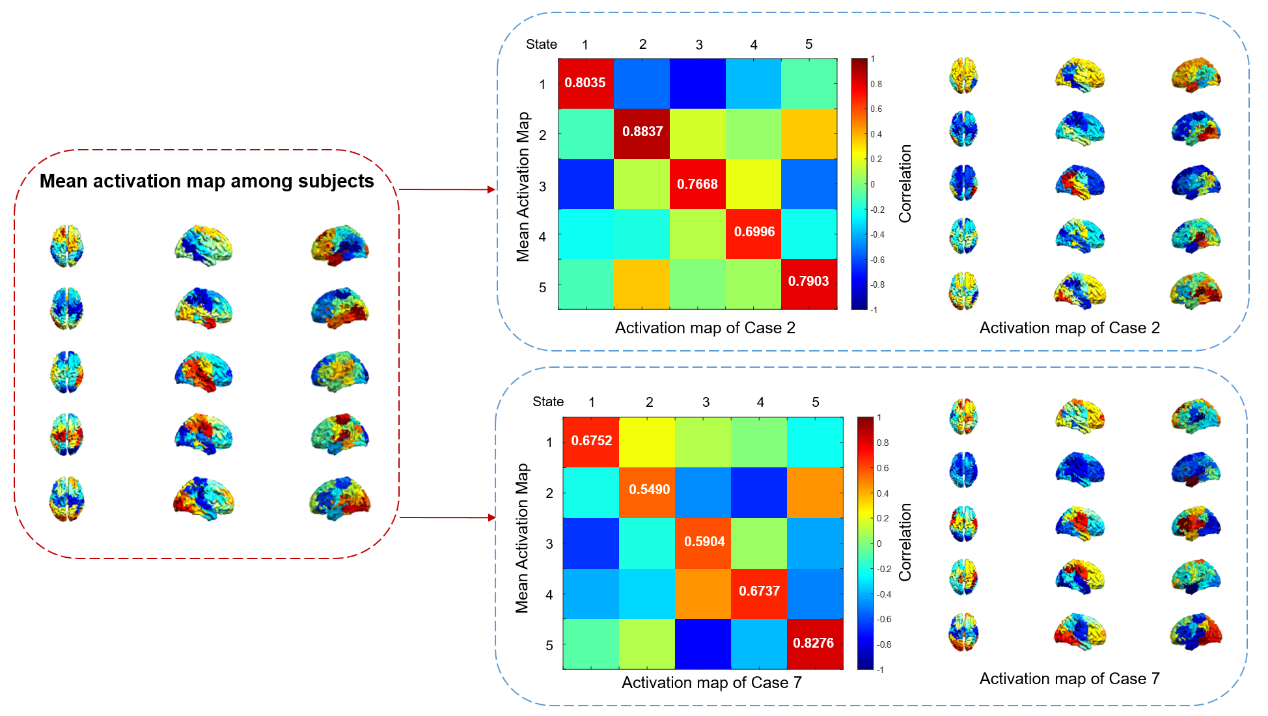


Supplementary Figure S3. The variability of subject-specific activation maps. Two subjects are shown: the best one (Case 2) at the top and a typical one (Case 7) at the bottom. Correlations with the mean activation map among all subjects are shown similarly to Figure 5.


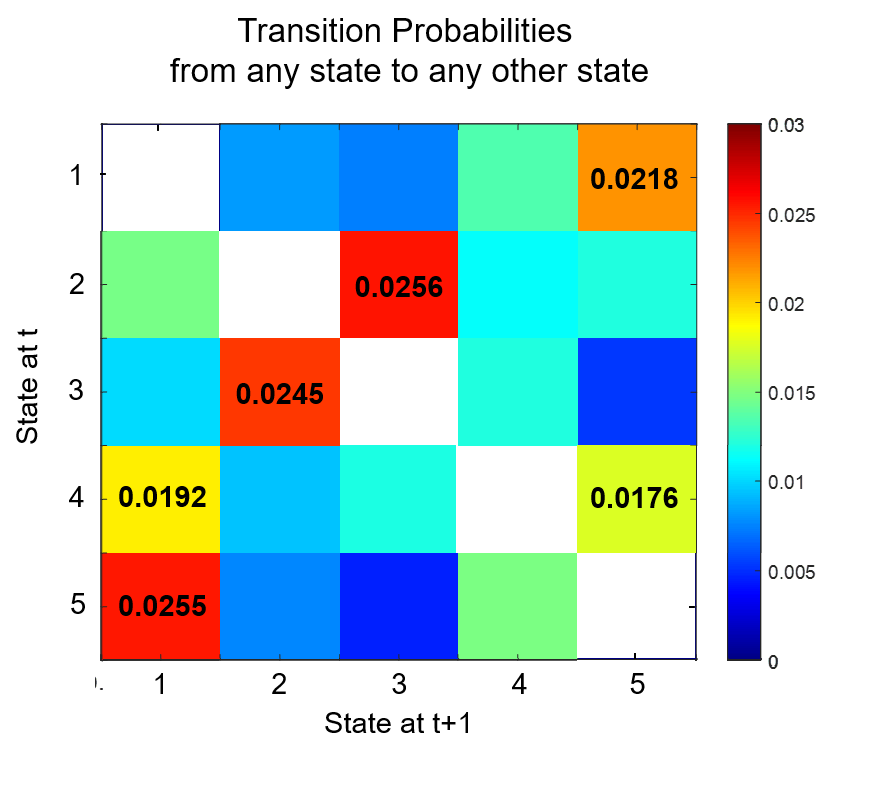


Supplementary Figure S4. The transition matrix of the HMM states.

Table S1

Maximum values in correlation spectra of the midpoints of electrodes with HMM states for all patients

*after FDR correction for the p values of correlation

| Subject Number | Midpoints of Electrodes MNI coordinates | Maximum correlation with | Maximum r values | Corresponding  f Hz |
| --- | --- | --- | --- | --- |
| Case1 | -37.1,-31.8,-16.6 | State2 | 0.2297* | 7.9 |
|  | 65.9,-19.4,-9.5 | State3 | 0.2179* | 6.6 |
|  | 36.8,-56.5,39.2 | State3 | 0.2019* | 11.4 |
|  | -28.3,-71.4,27.7 | State3 | 0.1795* | 13.3 |
| Case2 | -32.3,-22.6,-18.5 | State2 | 0.1439* | 7.3 |
|  | 30.5,-18.5,-19.0 | State3 | 0.1535* | 8.9 |
| Case3 | 43.3,-23.3,0.67 | State3 | 0.1672* | 8.6 |
|  | 35.1,-16.7,-14.8 | State3 | 0.0711 | 14.6 |
|  | 54.5,-37.7,-10.1 | State3 | 0.0874 | 13.6 |
|  | 44.5,-40.7,29.0 | State3 | 0.1387* | 7.5 |
| Case4 | 51.5,-2.0,12.1 | State2 | 0.1201 | 8.9 |
|  | 29.2,-31.4,69.2 | State3 | 0.0912 | 10.9 |
|  | 35.3,-34.3,64.6 | State1 | 0.0840 | 10.5 |
|  | 45.3,4.6,3.6 | State3 | 0.1136* | 11.5 |
| Case5 | -56.4,-27.9,-4.0 | State2 | 0.0940 | 5.4 |
|  | -47.5,-46.5,10.6 | State3 | 0.0972 | 5.2 |
|  | -40.3,-26.7,61.5 | State2 | 0.0679 | 2.1 |
|  | -30.9,-15.5,-12.2 | State2 | 0.0520 | 4.7 |
| Case6 | 40.9,-26.1,-14.4 | State3 | 0.1209 | 7.8 |
|  | 36.7,-16.7,60.9 | State1 | 0.0950 | 12.6 |
|  | 47.0,-30.5,50.2 | State1 | 0.0689 | 12.2 |
| Case7 | -59.9,-15.2.-22.0 | State3 | 0.2325* | 10.1 |
|  | 38.8,-6.6,-26.1 | State3 | 0.2734* | 12.1 |
|  | -47.1,-16.5,-21.9 | State3 | 0.2774* | 10.2 |
|  | -43.2,-10.3,12.7 | State3 | 0.2344* | 11.9 |
|  | -40.6,-2.2,6.2 | State3 | 0.1207* | 11.4 |
| Case8 | -45.9,-27.4,-8.6 | State2 | 0.1957* | 7.4 |
|  | -48.2,-23.0,-5.2 | State2 | 0.1019* | 5.7 |
|  | -59.3,-24.9,-6.2 | State2 | 0.1981* | 3.2 |
|  | -15.9,-69.3,30.5 | State2 | 0.1134* | 3.0 |
|  | -15.8,-78.7,11,1 | State5 | 0.0960 | 10.6 |
|  | 41.7,-24.8,-5.7 | State3 | 0.2917* | 10.2 |
| Case9 | -34.4,-20.9,-12.9 | State2 | 0.0854 | 8.4 |
|  | -13.3,41.4,32.1 | State3 | 0.0866 | 10.7 |
|  | -33.5,31.7,12.7 | State3 | 0.0883 | 10.1 |
|  | -12.8,-56.9,27.2 | State2 | 0.0823 | 14.1 |
|  | -13.5,-47.1,67.4 | State1 | 0.0498 | 9.3 |
|  | -49.9,1.7,5.9 | State5 | 0.0690 | 12.4 |
| Case10 | -57.1,-8.2,-12.3 | State2 | 0.1992* | 5.7 |
|  | -42.8,-42.9,-6.9 | State2 | 0.1183* | 4.9 |
|  | -9.1,37.8,36.1 | State1 | 0.0857 | 15.3 |
|  | -19.0,-47.7,59.4 | State5 | 0.0845 | 11.4 |
| Case11 | -47.3,-17.7,-16.5 | State2 | 0.2726* | 7.3 |
|  | -41.7,-2.6,-20.9 | State2 | 0.2430* | 7.4 |
|  | -48.6,-37.2,-8.4 | State2 | 0.1413* | 3.1 |
|  | -20.7,-83.4,-9.5 | State5 | 0.1165* | 12.6 |
|  | -29.2,-87.9,15.3 | State5 | 0.1159* | 11.4 |
|  | -21.7,-59.7,24.2 | State3 | 0.0791 | 11.1 |
|  | -24.8,-46.1,35.4 | State5 | 0.0923 | 12.2 |
|  | 35.3,-16.4,-22.8 | State3 | 0.1056* | 7.9 |
